# Activities and impacts of patient engagement in CIHR SPOR funded research: a cross-sectional survey of academic researcher and patient partner experiences

**DOI:** 10.1186/s40900-022-00376-4

**Published:** 2022-08-29

**Authors:** Anna Maria Chudyk, Roger Stoddard, Nicola McCleary, Todd A. Duhamel, Carolyn Shimmin, Serena Hickes, Annette S. H. Schultz

**Affiliations:** 1 College of Nursing, Rady Faculty of Health Sciences, University of Manitoba, CR3024 - 369 Tache Avenue, Winnipeg, MB R2H 2A6 Canada; 2 Horizon Health Network, 80 Woodbridge Street, Fredericton New Brunswick, E3B 4R3 Canada; 3 Clinical Epidemiology Program, Ottawa Hospital Research Institute, Room L1202, 501 Smyth Road, Box 711, Ottawa, ON K1H 8L6 Canada; 4 School of Epidemiology and Public Health, University of Ottawa, 600 Peter Morand Crescent, Ottawa, ON K1G 5Z3 Canada; 5 Faculty of Kinesiology and Recreation Management, 212 Active Living Centre, Winnipeg, MB R3T 2N2 Canada; 6 George and Fay Yee Centre for Healthcare Innovation, 3rd floor – 753 McDermot Avenue, Winnipeg, MB R3E 0T6 Canada; 7 Translating Emergency Knowledge for Kids (TREKK) Parent Advisory Group, Children’s Hospital Research Institute of Manitoba, 512E - 715 McDermot Avenue, Winnipeg, MB R3E 3P4 Canada; 8 College of Nursing, Rady Faculty of Health Sciences, University of Manitoba, CR3022 - 369 Tache Avenue, Winnipeg, MB R2H 2A6 Canada; 9 Institute of Cardiovascular Sciences, St. Boniface General Hospital Albrechtsen Research Centre, Winnipeg, Canada

**Keywords:** Patient and public involvement, Patient involvement, Stakeholder engagement, Patient engagement in research, Patient-oriented research

## Abstract

**Background:**

Knowledge about the specific engagement activities pursued and associated impacts of patient engagement in research in Canada remains nascent. This study aimed to describe engagement activities and perceived impacts of projects funded by the Strategy for Patient-Oriented Research (SPOR).

**Methods:**

This was a cross-sectional online survey of academic researchers and patient partners engaging in projects funded through 13 SPOR funding calls (2014–2019). Patient engagement activities and impacts were measured using a self-developed survey. Thematic analysis was used to describe engagement activities and impacts.

**Results:**

66 of 511 academic researchers and 20 of 28 patient partners contacted completed the survey and were included in analyses. Respondents reported that patient partners were engaged in seven types of activities across the research cycle: (a) sharing experiences/giving advice, (b) identifying the research focus/methods, (c) developing/revising aspects of the project, (d) conducting research activities, (e) study participation, (f) presenting on behalf of the project, and (g) other grant development or knowledge translation activities. Engagement was associated with six different types of impacts related to knowledge, outputs, or directions being (a) created, (b) moulded, (c) confirmed, or (d) chosen/prioritized, (e) perceived success of the research, and (f) minimal/negative impacts on the research.

**Conclusions:**

This study presents information on different ways that patient partners were engaged in SPOR-funded research and the potential impacts of these activities. This knowledge base is imperative to the future of patient engagement in research, including the planning and evaluation of future studies that engage patients as active shapers of research.

**Supplementary Information:**

The online version contains supplementary material available at 10.1186/s40900-022-00376-4.

## Plain English Summary

The Canadian Institutes of Health Research developed the Strategy for Patient-Oriented Research (SPOR) to help increase capacity for patient engagement in research. However, little is known about the ways in which Canadian patient co-researchers (i.e., patient partners) are being engaged in research and the perceived impacts of engagement. Therefore, this study aimed to describe engagement activities and perceived impacts of SPOR-funded projects. To do so, we carried out an online survey of academic researchers and patient partners engaging in projects funded through 13 SPOR funding calls. We analysed the collected data using thematic analysis, which focuses on finding themes among data. Sixty-six of 511 academic researchers and 20 of 28 patient partners contacted completed the survey and were included in analyses. We found that patient partners were engaged in seven types of activities across the research cycle: (a) sharing experiences/giving advice, (b) identifying the research focus/methods, (c) developing/revising aspects of the project, (d) conducting research activities, (e) study participation, (f) presenting on behalf of the project, and (g) other grant development or knowledge translation activities. We also found that engagement was associated with six different types of impacts related to knowledge, outputs, or directions being (a) created, (b) moulded, (c) confirmed, or (d) chosen/prioritized, (e) perceived success of the research, and (f) minimal/negative impacts on the research. The findings of this study can be used to inform ongoing and future research, including empowering patient partners to be more informed and actively shape how they may contribute to research processes.

**Supplementary Information:**

The online version contains supplementary material available at 10.1186/s40900-022-00376-4.

## Background

The active engagement of patients and informal caregivers (e.g., families or friends) as co-producers of research, known as patient engagement in research, is increasingly recognized as a cornerstone of health research. Patients and caregivers are the public funders of research, and directly affected by its processes and outcomes. Thus, there is a moral obligation to involve these stakeholders in research design and conduct [1]. Patients and caregivers also possess experiential knowledge of living with a health condition or accessing healthcare services that is unique and complementary to the scientific knowledge possessed by academic researchers and clinicians [2]. Consequently, global research institutions such as INVOLVE (United Kingdom) and the Patient-Centered Outcomes Research Institute (PCORI, United States) have been established to champion and fund patient engagement in research. In Canada, the Canadian Institutes of Health Research (CIHR) developed the Strategy for Patient-Oriented Research (SPOR) to help increase capacity for patient engagement in research and transform the traditional role of the patient and caregiver from passive participant to active shaper of research and, subsequently, health care [3].

As interest in patient engagement has grown among health researchers, so has the focus on better understanding different approaches to patient engagement in research and their resulting impacts, especially among researchers in the United Kingdom and USA [4, 5]. Given the multitude of ways (e.g., at different points in the research cycle, through different activities) and levels (e.g., passive study participant, consultant providing feedback and advice, collaborator that is an equal study partner) [6, 7] that patients and caregivers can be engaged in research, this information is useful to researchers in planning and conducting future studies and reflecting on their current engagement practices. This knowledge can also help patients and caregivers gain more awareness about the ways that they can contribute as co-researchers and the types of influence they may have. Since collective knowledge about the specific engagement activities pursued and resulting impacts of patient engagement in research in Canada remains nascent [4, 7], we conducted a cross-sectional survey that aimed to describe engagement activities and perceived impacts of SPOR-funded research. To our knowledge, this is the first published Pan-Canadian study to gather primary data on the activities and impacts of patient engagement from the perspectives of both academic researchers and patient and caregiver co-researchers (herein referred to as patient partners).

## Methods

### Guiding framework

**Fig. 1:**
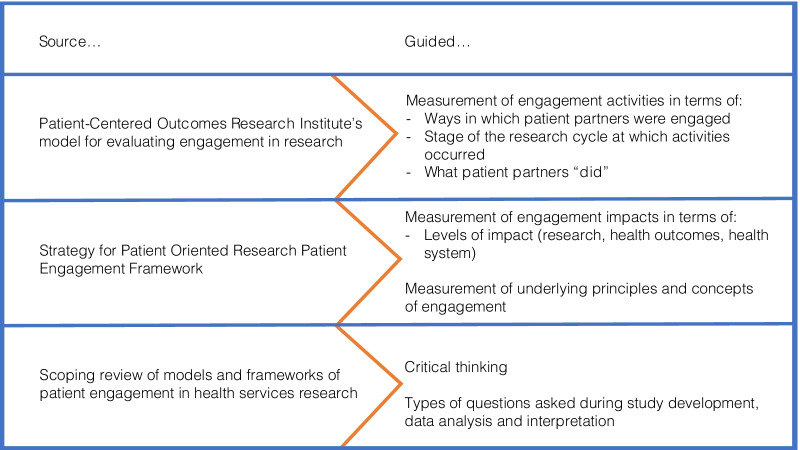
Overview of the study’s guiding framework

This study’s conceptualization of patient engagement in research was guided by SPOR Patient Engagement Framework [8], PCORI’s model for evaluating engagement in research [9], and our scoping review of models and frameworks of patient engagement in health services research [10, 29] (Fig. 1). SPOR’s framework includes key concepts, guiding principles, and desired impacts (i.e., on the research process, improving health outcomes, and enhancing the health system) of patient engagement in research [8]. It does not, however, conceptualize the activities that underlie patient engagement. Thus, we drew on PCORI’s model in describing patient engagement activities, in particular stages of research at which engagement occurred, and the type of activities that occurred (i.e., what patient partners “did”) [9]. Finally, our scoping review guided our thinking and the types of questions we asked when analyzing and interpreting our data [10].

### Setting and study design

This cross-sectional online survey targeted academic researchers and patient partners engaging in SPOR-funded projects across Canada. It was the first study within a three-part project that also explored the engagement-related experiences of patient partners who completed this survey and consented to further participation in a qualitative interview, and a multi-day virtual workshop that explored the current and preferred future states of Canadian patient engagement in research from the perspectives of SPOR-funded academic researchers and patient partners. Qualtrics, an online survey platform, was used to manage recruitment and administer the survey. Collected data were stored on a secured network server at the first authors’ (AMC) institution. The Checklist for Reporting Results of Internet E-Surveys [11] and the Guidance for Reporting Involvement of Patients and the Public checklist-short form [12] guided reporting. Ethics approval was obtained from the Education Nursing Research Ethics Board at the University of Manitoba (certificate number E2019:082(HS23180)).

### Participants

Our sampling frame consisted of academic researchers and patient partners engaging in projects funded through 13 SPOR funding calls (2014–2019, Additional file: 1, 2). Academic researchers were identified through listings of successfully funded principal applicants in CIHR’s publicly available Funding Decisions Database. Those without publicly available email addresses were excluded. As there is no repository of patient partners engaged in SPOR-funded projects, patient partners were identified through snowball sampling, including social networks (i.e., sharing the recruitment poster on Twitter), professional networks (i.e., sharing a study overview and the recruitment poster with our local SPOR SUPPORT Unit and study team members’ colleagues via email and asking them to disseminate widely), and by asking sampled academic researchers to share our study information with patient partners they engaged with and/or providing us with patient partners’ contact information so that we could follow-up with them directly.

### Recruitment

Eligible academic researchers and identified patient partners were sent recruitment emails that contained a study overview, consent form (providing details such as the study purpose and investigators, estimated survey completion time, and data storage protocols), and a personalized link to a closed survey. Due to the widespread impact of the COVID-19 pandemic, an a-priori decision was made to hold two separate recruitment rounds (i.e., April 14–June 8 and October 22–December 15, 2020) to provide those who were potentially unable to take part in the first round due to the pandemic further opportunity to participate. Only individuals that did not complete a survey in the first round were contacted during the second round. Informed consent was inferred through voluntary completion of the survey. Participants chose whether to receive a $5 e-gift card or have the study make a $5 donation to the Canadian Cancer Society on their behalf.

### Survey design and administration

There is no “gold standard” survey to measure patient engagement activities and impacts. The majority of tools for evaluating patient engagement in research do not have established psychometric properties and do not measure the perspectives of both patient partners and academic researchers [13]. Thus, we applied design methods proposed by Dillman et al. [14] and our study’s guiding framework (Fig. 1) to develop a survey that measured activities and perceived impacts of patient engagement in research. The survey (Additional file: 1, 2) included modified items drawing from PCORI’s evaluation of patient engagement in research [9] and newly created items that measured the elements identified within SPOR Patient Engagement Framework [8], participants’ sociodemographic characteristics and characteristics of their SPOR-funded project. This current study only reports on data from open-ended items asking respondents to describe what patient partners did and the influence/impact it had on project decisions/processes, by stage of the research cycle.

Academic researchers and patient partners completed separate survey versions which contained conceptually similar items. However, academic researchers reported on engagement activities across their entire SPOR-funded project, whereas patient partners only reported on engagement activities they were involved in. Adaptive questioning (i.e., skip logic) was used to reduce the number and complexity of questions. Participants were able to review and change their answers prior to survey submission. To help prevent duplicate entries from the same respondent, individuals were sent personal survey links which could not re-access the survey once it was completed. The survey’s content validity was established by mapping items across the dimensions of the study’s guiding framework, while its face validity was assessed among the research team (composed of two patient partners and four academic researchers). The usability of the survey’s virtual administration mode (i.e., Qualtrics) was piloted among the research team and six colleagues (none of whom were eligible to participate in the study).

### Patient engagement

Two patient partners (RS and SH) were engaged throughout the study at the level of involve [6, 7]—that is, they were consistently engaged as research team members throughout the study, with their input and perspectives being used to inform the study decisions that were ultimately made by the first and senior author. They primarily provided ideas and feedback during small group or full team meetings and helped revise study documents. These activities contributed to developing the study’s underlying grant, designing and testing the survey and study protocol, shaping data analysis and interpretation, and developing this manuscript. Factors supporting the meaningful influence of these activities on study directions included patient partners’ early involvement, a co-developed terms of reference and an engagement liaison that guided engagement activities, and mindful attention to the relational aspects of engagement.

### Data analysis

Descriptive statistics (medians (25th and 75th percentiles) and counts (percentages)) were used to summarize sociodemographic and project characteristics. Thematic analysis [15] was used to describe patient engagement activities and impacts. Theme development was directed by the study’s guiding framework and led by the first author (AMC), who performed the initial coding and then consulted with two other co-authors (RS and AS) via multiple meetings in which they discussed how the individual responses fit together to represent the emergent themes. The results from the work of these three co-authors were then shared with the full team, who had the opportunity to help refine the themes during a half-day meeting focused on data analysis and the iterative process of developing this manuscript. Given the presence of value-based responses about the impacts of engagement, Aubin et al.’s framework for measuring the impact of patient-oriented research was also applied to classify benefits and advantages to patient partners and academic researchers as “value to patients” (e.g., increased research knowledge, feeling empowered) and “value to academic researchers,” (e.g., improved understanding of a health condition from the patient perspective, new research scope or opportunities) respectively [16]. Descriptive statistics were performed using IBM SPSS Statistics 27, and thematic analysis was managed through Nvivo (v.12.6.0).

## Results

### Participant flow into the study

**Fig. 2:**
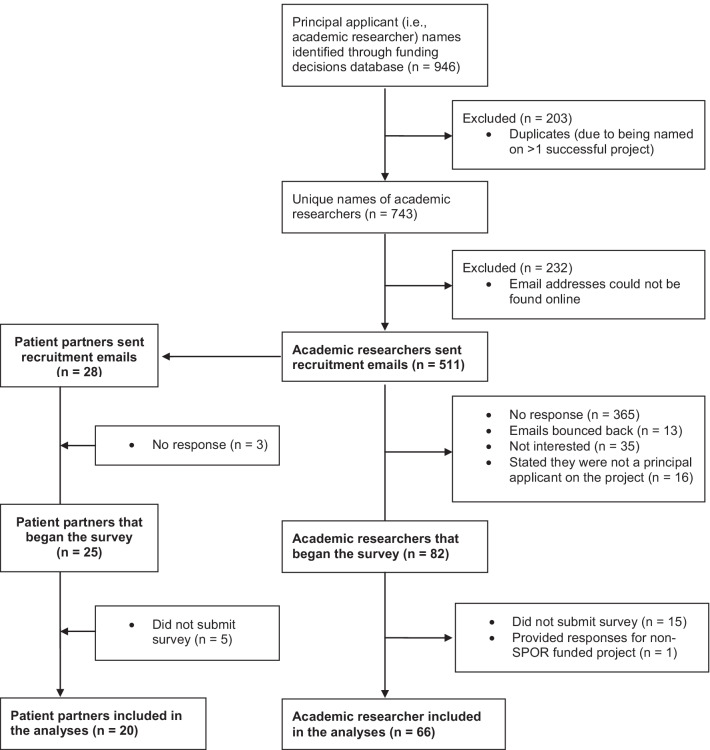
Flow of participants into the study

Figure 2 presents the flow of participants into the study. Of the 511 principal applicants sent a recruitment email, 82 (16%) filled in at least the first survey page, and 67 completed the survey, all representing the perspectives of academic researchers. Of these respondents, one answered survey questions in relation to a non-SPOR funded project. Thus, responses from 66 (13%) academic researchers were included in the analyses. In addition, of the 28 patient partners sent a survey link, 20 (71%) completed the survey and were included in analyses.

### Participants’ sociodemographic characteristics

As displayed in Table 1, the majority of academic researchers and patient partners self-identified as female (66% and 75%, respectively), of Caucasian/European ancestry (82% and 80%, respectively), and residing in Ontario (42% and 50%, respectively). Seventy-five percent and 25% of patient partners stated they represented the patient and caregiver perspectives, respectively. Approximately 75% of patient partners had an undergraduate or graduate university degree. A Wordcloud of academic researchers’ departments/disciplines is presented in Additional file 3.

**Table 1:** Participants’ sociodemographic characteristics, by group

	Academic researchers, n (%)	Patient partners, n (%)
Age, years	45 (42, 50)^a,b^	60 (48.5, 66)^b,c^
Gender	20 (31%) Male	4 (20%) Male
	43 (66%) Female	15 (75%) Female
	2 (3%) Prefer not to answer	1 (5%) Prefer not to answer
	1 Missing	
Ancestry	53 (82%) White/Caucasian/European	16 (80%) White/Caucasian/European
	5 (8%) South Asian	1 (5%) South Asian
	3 (5%) Mixed Ethnicity	
	3 (5%) Other	
		2 (10%) First Nations/Inuit/Metis
		1 (5%) East Asian
	2 Missing	
Place of residence (province/territory)	8 (12%) Alberta	3 (15%) Alberta
	8 (12%) British Columbia	3 (15%) British Columbia
	6 (9%) Manitoba	3 (15%) Manitoba
	2 (3%) New Brunswick	
	2 (3%) Newfoundland and Labrador	
	3 (5%) Nova Scotia	
	27 (42%) Ontario	10 (50%) Ontario
	8 (12%) Quebec	1 (5%) Quebec
	1 (2%) Saskatchewan	
	1 Missing	
Highest level of education completed	N/A	2 (10%) Completed secondary school
		3 (15%) Completed trade/technical school or college diploma
		6 (30%) Completed university degree
		9 (45%) Completed graduate degree
Primary community represented	N/A	15 (75%) Patient/consumer
		5 (25%) Caregiver

^a^n = 59

^b^median (25th and 75th percentile)

^c^n = 20

### Activities and impacts of engagement

Patient partners were engaged in seven types of activities across the research cycle. These included: (a) sharing experiences or giving advice, (b) helping identify or choose the research focus or methods, (c) helping develop or revise aspects of the grant, study, or knowledge translation outputs, (d) helping conduct research activities, (e) participating in the study, (f) presenting on behalf of the study, and (g) other grant development or knowledge translation activities. It should be noted that any reported study participation activities (g) occurred in addition to a given project’s other types of engagement activities.

Tables 2, 3, 4, 5 and 6 present more detailed data on how these seven types of patient engagement activities were enacted across the research cycle, as well as their perceived impacts. As reflected in these tables, participants reported six different types of impacts related to knowledge, outputs, or directions being (a) created, (b) moulded, (c) confirmed, or (d) chosen/prioritized, (e) perceived success of the research, and (f) minimal or negative impacts on the research (see Additional file: 4 for the terms encompassed by each impact type). Among the 95 impacts noted in these tables, 76 were related to research processes. The remaining seven related to academic researcher values (i.e., initiating new project directions based on patient partner insights, gaining a deeper understanding of the patient experience), eight to patient partner values (i.e., increased confidence among the research team, greater sense of ownership over the study and its findings, research experience, broadened thinking around patient engagement in research), and four to impacts on health outcomes or health systems (i.e., strengthened impact of the study findings, increased richness of patient experience within health systems). Lastly, although 86/95 reported impacts resulted in perceived beneficial changes, nine reported impacts were perceived as minimal or having a negative influence on the research.

**Table 2:**
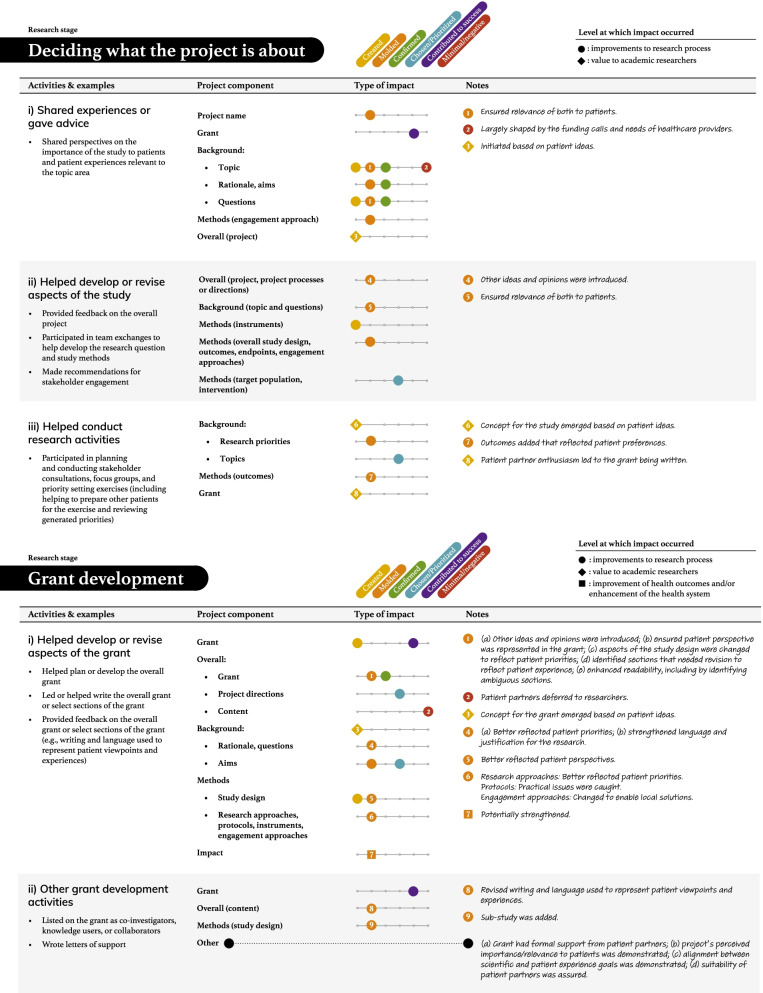
The activities and impacts of patient partners in helping decide what the project is about and grant development

**Table 3:**
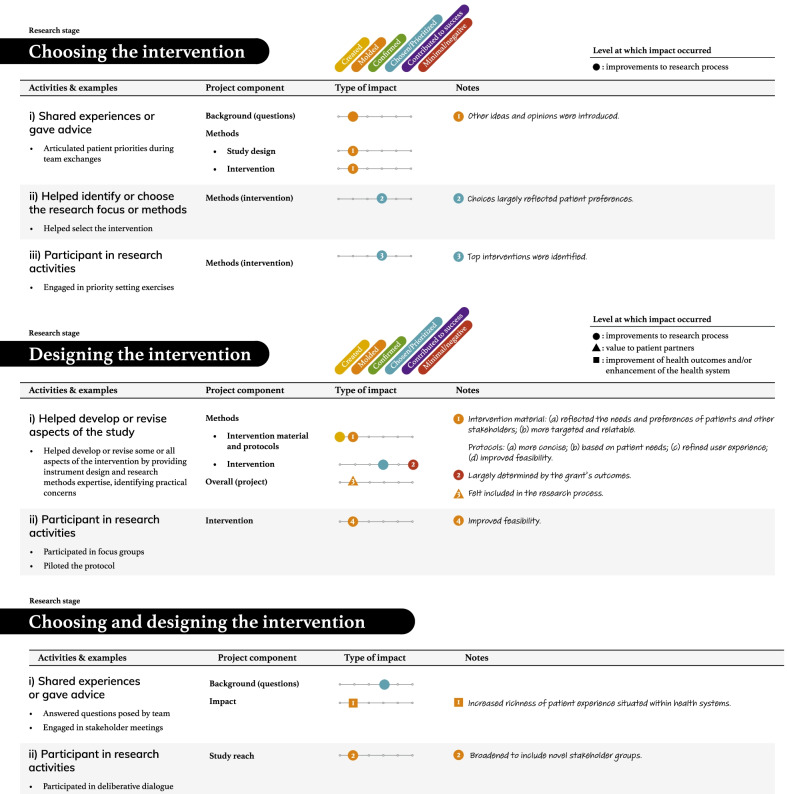

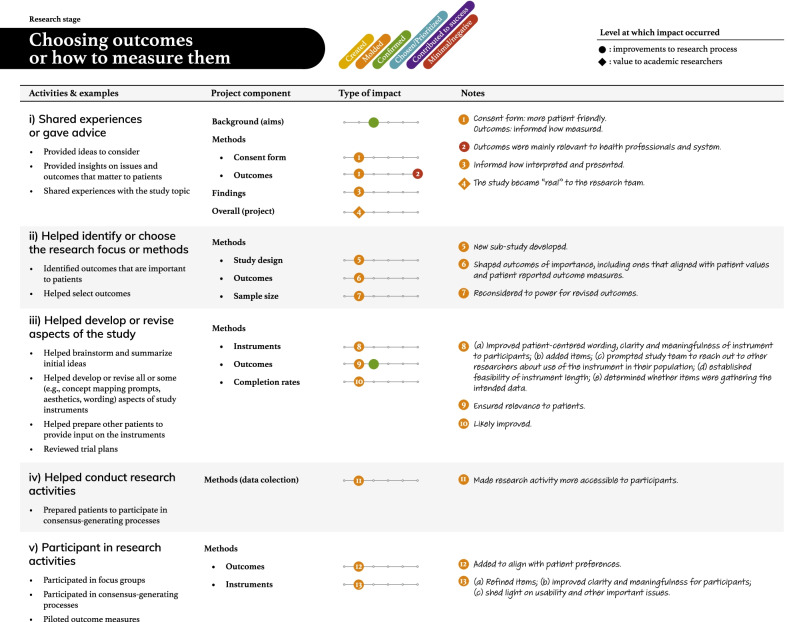
The activities and impacts of patient partners in intervention and outcome design

**Table 4:**
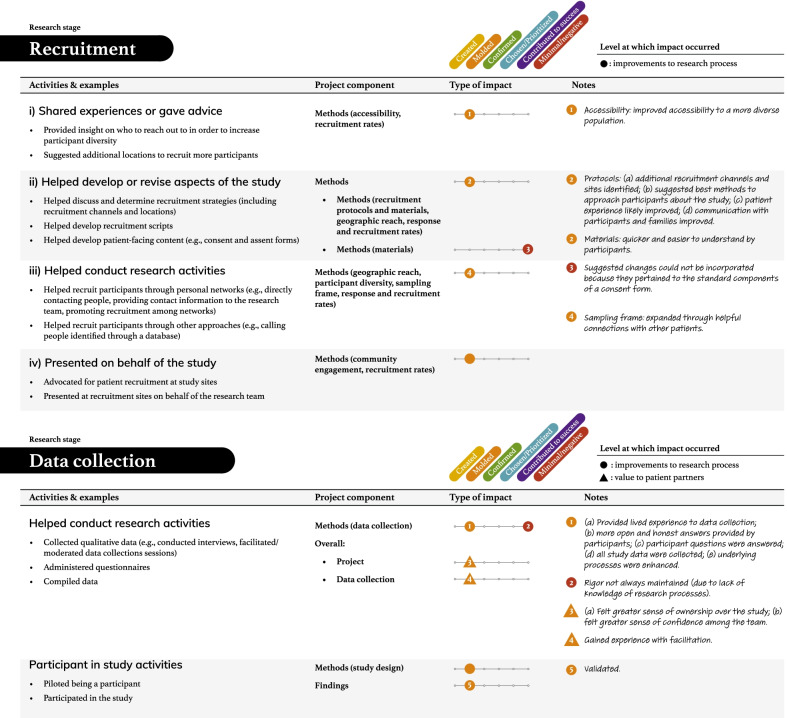
The activities and impacts of patient partners in recruitment and date collection

**Table 5:**
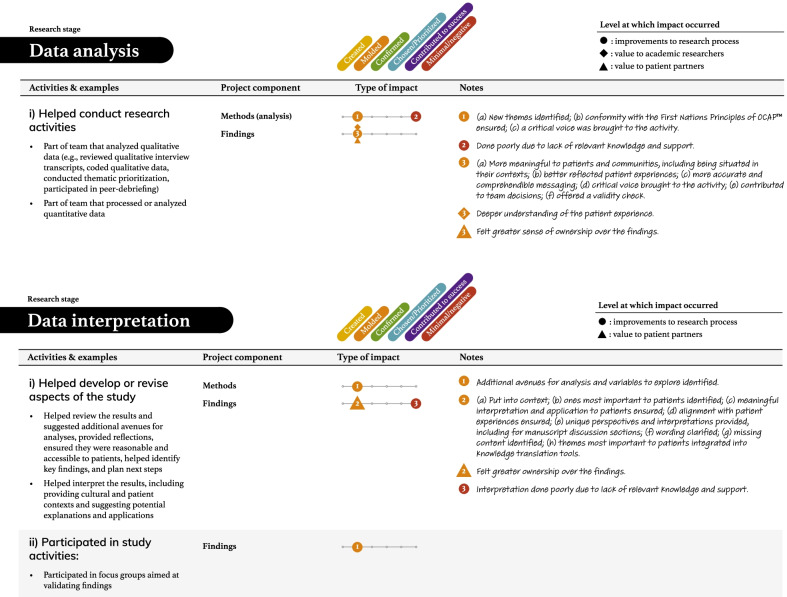
The activities and impacts of patient partners in date analysis and interpretation

**Table 6:**
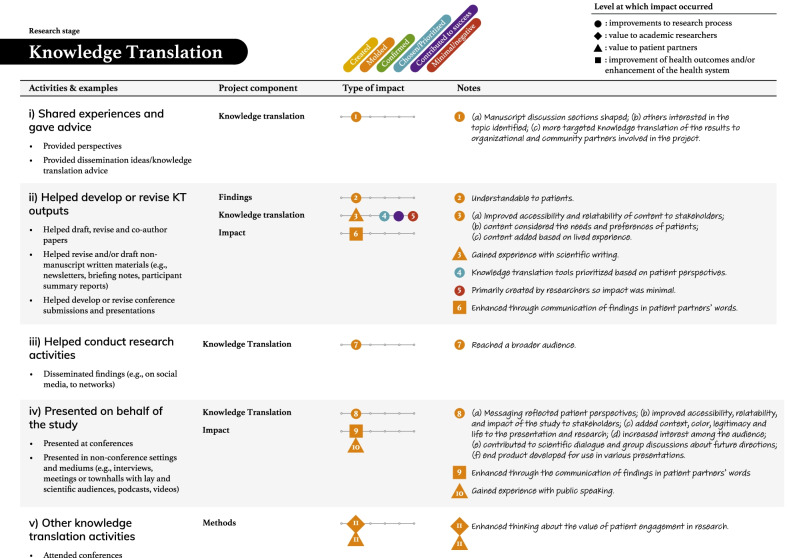
The activities and impacts of patient partners in knowledge translation

## Discussion

This study found that the diversity in which patient partners were engaged across the research cycle could be organized into seven over-arching categories. Further, engagement was associated with six different types of impacts, which predominantly led to perceived improvements in research processes. The direct applications of study findings to patient engaged research are detailed below.

Patient engagement in research encompasses a spectrum of activities, defined by the direction of information flow and decision-making power between academic and patient co-researchers [6, 7]. While this conceptual fluidity offers diverse possibilities, the unintended consequence is confusion among academic researchers [19, 20] and patient patient partners about the practicalities of how and to what extent patients can contribute to the research process. The present study addresses this uncertainty by mapping different ways that patient partners contribute to research. We envision this mapping informing ongoing and emerging patient engaged research, including initial (e.g., terms of reference) and long-term (e.g., manual of procedures) engagement plans. Importantly, as patient partners typically have less research-related training and experience, this knowledge can also help shift power balances by helping them be more informed and actively shape how they will contribute to research processes. Lastly, there is a lack of practical guidance in the peer-reviewed published literature on how to engage patients in research [18]. The majority of this practical guidance comes from grey literature publications such as those published by SPOR-affiliated entities (see for example [21–23]) and other research bodies and organizations (see for example [24, 25]). This study provides peer-reviewed findings that can be used to advance and validate knowledge of engagement activities suggested in these reports.

Over the last six years, there has been an influx in studies investigating the impacts of patient engagement in research, especially in the United Kingdom and USA [4]. Relatively few studies have investigated the impacts of patient engagement in Canada [4], which is problematic because these local data are important for building the evidence base to support the continued investment of Federal funding into patient engagement initiatives and organizations like SPOR and to evolve current engagement practices. Further, lack of impact-related knowledge limits the decisions that researchers make when designing studies and evaluating patient engagement [26]. Knowledge of the impact types reported in this study, and their examples across the research cycle, can be directly applied by academic researchers and patient partners to: (a) reverse engineer engagement plans and incorporate specific prompts that target desired areas of impact, (b) inform the evaluation of patient engagement activities, and (c) provide those who are hesitant to adopt this approach or uncertain about how they can contribute with ideas on potential areas of influence. These data can also contribute to existing Canadian efforts to develop a unified framework for measuring the impact of patient engagement in research [16, 28] by identifying potential perceived impacts of engagement that can inform or validate the ensuing framework.

Current evidence tends to focus on the impact of patient engagement within the research process, including facilitating recruitment and study enrollment, contributing to data collection and analysis, and dissemination and presentation of study findings [4, 17, 18]. This is not surprising given the biomedical interests underlying much of health research. While the majority of identified impacts influenced research processes, we also identified impacts related to personal values. However, these represented a smaller minority of reported impacts than expected based on previous work [4, 18]. For example, in a recent scoping review of scoping reviews, Modigh et al. found four different over-arching types of positive impacts on patients that engaged in research, including developing new skills and knowledge (e.g., research, teamwork), personal development (e.g., increased confidence and self-esteem), support and friendship (e.g., receiving and giving support), and enjoyment and satisfaction (e.g., feeling valued, making a contribution) [4]. They also reported three positive over-arching academic researcher specific impacts, including improved knowledge and understanding of the community (e.g., identifying new issues, greater understanding of the patient perspective), enjoyment and satisfaction, and challenges to beliefs and attitudes (e.g., challenged prejudices, changed expectations and assumptions). Our finding of a limited amount of value-based impacts may be biased by the wording of the items we used to measure impact. It may also be influenced by the fact that SPOR’s patient engagement framework conceptualizes patient engagement-related outcomes as existing at the levels of the research process, health outcomes, and health systems [8]. As SPOR is the study sample’s funding body, respondents may have been more likely to consider these levels of impact when reflecting on the impacts of engagement. Fortunately, work that builds upon this patient engagement framework has called attention to the need to expand the focus to incorporate the personal values that patient partners and academic researchers derive from engagement [16].

Our study advances the knowledge base concerning ways to engage patient partners across the research cycle and the impacts of these engagement activities. However, we acknowledge that many factors affect whether engagement activities achieve desired impacts, such as where engagement activities are situated along the spectrum of engagement [6, 7] and the dynamics of the research team and its encompassing environment. Future studies should incorporate experimental designs or investigate the steps needed for future research to support causal inferences being drawn about the impacts of engagement from observational data. This work will serve to both better guide research partners on how to engage with each other to achieve desired impacts and contribute hard evidence to support the benefits of patient engagement in research. Another interesting line of inquiry would be to directly compare patient partners’ and academic researchers’ characterizations and perceptions of the engagement related activities and impacts they were involved in when partnering on the same study. Similarities and differences between their responses could yield novel insights into the nature of engagement experiences.

Our study has some limitations. The 13% response rate for academic researchers and undefined sampling frame for patient partners undermines the generalizability of study findings. However, this study is not meant to present an evaluation of how patient engagement is being carried out by SPOR-funded researchers. Rather, it is intended to present information on different ways that patient partners are being engaged in SPOR-funded research and the potential impacts of these engagement activities. Other factors that may affect the generalizability of our study findings include our focus on Canadian health researchers (academic and patient) funded through SPOR and the lack of diversity among study participants. It would be helpful if funding bodies such as SPOR gathered publicly available data that measured diversity-related characteristics of grant recipients so as to support determining whether this limitation resides at the level of the study and/or system. Social desirability bias may have resulted in respondees providing more positive responses about engagement impacts. We tried to limit this through the collection of anonymized data. Finally, our survey assumed reported impacts of engagement were perceived, which means a definitive casual link cannot be drawn between the patient engagement activities and impacts reported.

## Conclusions

There is a growing interest in patient engagement in research among researchers and funding agencies in Canada and internationally. However, relatively little is known about how patients are engaged and the impacts of the activities in Canadian research. Our study advances knowledge of patient engagement in research by providing practical evidence to address this gap. This knowledge base is imperative to the future of patient engagement in research, which includes the planning and evaluation of future studies that engage patients as active shapers of research, and subsequently, health care.

## Supplementary Information

**Additional file 1**. Survey of engagement-related activities and impacts: Patient partner version.

**Additional file 2**. Survey of engagement-related activities and impacts: Academic researcher version.

**Additional file 3**. Wordcloud of academic researcher respondents’ departments/disciplines.

**Additional file 4**. Terms encompassed by each reported impact type.

## Data Availability

The datasets used and/or analyzed during the current study are available from the corresponding author on reasonable request.

## Abbreviations

CIHR: Canadian Institutes of Health Research
PCORI: Patient-Centered Outcomes Research Institute
SPOR: Strategy for Patient-Oriented Research
