# Supplementary material for: Activities and impacts of patient engagement in CIHR SPOR funded research: a cross-sectional survey of academic researcher and patient partner experiences
Source: Res Involv Engagem. 2022 Aug 29;8:44. doi: 10.1186/s40900-022-00376-4 (PMC9423700; doi:10.1186/s40900-022-00376-4)
Supplement: Supplementary file 1 — Additional file 1. Survey of engagement-related activities and impacts: Patient partner version. [file 40900_2022_376_MOESM1_ESM.pdf]

Supplementary File 1

Please note, as described in our manuscript, this survey contains modified items drawing from PCORI's evaluation of patient engagement in research (i.e., Ways of Engaging-ENgagement ACtivity Inventory Tool (We-ENACT)) and newly created items that measured the elements identified within SPOR Patient Engagement Framework, participants' sociodemographic characteristics and characteristics of their SPOR-funded project.

Survey items were distributed across 7 pages. Technology-assisted completeness checks were not performed prior to survey submission.

Overview

Welcome! The purpose of this online survey is to gather information about the activities and impacts of patient engagement in research projects funded through Strategy for Patient-Oriented Research (SPOR). This online survey should take approximately 15-20 minutes to complete and your participation is entirely voluntary.

Here is a list of the acronyms that you will come across when completing the survey:

iCT: Innovative Clinical Trial
JDRF: The name of a non-profit organization that funds type 1 diabetes research
PIHCI: Primary and Integrated Health Care Innovations
SPOR: Strategy for Patient-Oriented Research

Thanks again for your time and participation!

Patient engagement in research: Who, how, and why

1. Are you comfortable completing this survey online or would you rather set up a time for us to call you so that you can give your answers over the phone?
    a. I'm ok to continue filling this survey out online!
    b. I'd rather set up a time for you to call me so that we can fill out the survey together over the phone

** If 1b (sent to a different IP location): Please provide your name and email or phone number. A member of our research team will contact you within 2 business days to set up a date and time for you to answer the survey by phone.

Patients can contribute to research projects in many ways. This could include:

- Making sure researchers know what kind of information is important to patients;
- Deciding what the study should be about;

- Deciding who to include in the study;
- Choosing what outcomes the study will measure;
- Tracking study progress;
- Sharing study findings.

We call contributing to research projects in ways like this being a "**patient partner**".

2. Are or were you a patient partner on a SPOR-funded research project? The funding calls for these projects took place between 2014 and 2019.
    a. Yes
    b. No → If you were a principal investigator (instead of a patient partner) on a SPOR-funded project, please click on the following link to be taken to the principal investigator version of the survey:

       [survey link]

       Otherwise, thank you for your interest in our survey but you do not meet our study's eligibility criteria.

A little background information on the project

You may be a patient partner on more than one research project funded through a SPOR funding call. However, you were sent this link because of your involvement in a specific project. Please **answer the following questions with only this project in mind**. We are interested in your experiences **since the project(s) began**.

3. **What is the name of the SPOR-funded project you are describing in this survey** (as listed on the successfully funded grant application)**?** If you're unsure, we included your project's name in the email we sent with the link to this survey. Since other members of your research team may be filling out this survey as well, we'd like to use this information to link your responses and get a full picture of the project's patient engagement activities and impacts.

4. What SPOR funding call supported this project?
    a. Catalyst Grant: SPOR Innovative Clinical Trials
    b. SPOR Networks in Chronic Disease
    c. Patient-Oriented Research Collaboration Grants
    d. Operating Grant: SPOR Primary and Integrated Health Care Innovations (PIHCI) Network – Programmatic Grants
    e. Operating Grant: SPOR PIHCI Network - Quick Strikes
    f. Operating Grant: SPOR PIHCI Network - Knowledge Synthesis Grants
    g. Operating Grant: SPOR PIHCI Network - Comparative Program and Policy Analysis
    h. Operating Grant: SPOR Innovative Clinical Trial (iCT) Multi-Year Grant
    i. Operating Grant: SPOR - Guidelines and Systematic Reviews
    j. Operating Grant: SPOR & JDRF - iCT Multi-Year Grant

k. Operating Grant: SPOR & JDRF - iCT Multi-Year Grant - Metabolic Control & Artificial Pancreas and Devices
l. Operating Grant: SPOR iCT Rewarding Success - Development Grants
m. Team Grant - SPOR iCT Rewarding Success - Phase 3
n. Other - *Please describe*
o. Don't know

5. What research phase is this project currently in?
   a. Planning how to carry out the project
   b. Finding patients to participate in the study (recruitment)
   c. Collecting data from study participants
   d. Analysing or reviewing findings (results)
   e. Writing-up/sharing study findings with those who can use them
   f. The project is done
   g. Other – *Please describe*
   h. I don't know

6. What is the main reason you wanted to contribute to the research project?

7. Please describe how you came to be involved as a patient partner (e.g., recruited) with this project.

8. Were you compensated for your contributions to this project (select all that apply)?
   a. Yes – financially (*please describe)*
   b. Yes – other (*please describe*)
   c. No

9. Some of the projects funded by SPOR consist of multiple studies. Does the research project you are describing consist of…
   a. A single study? → skip to question 12
   b. Multiple studies?
   c. I don't know → skip to question 12

10. Were <u>you</u> involved across all of the studies (entire project) or some of the studies (a portion of the project)?
   a. All of the studies (entire project)
   b. Some of the studies (portion of the project)
   c. I don't know

11. Were other patient partners involved in the other studies (parts of the project) that you <u>weren't</u> involved in?
   a. Yes
   b. No
   c. I don't know

Patient engagement activities and impacts

12. There are many ways that patient partners can be engaged in a research project. Please select all of the ways that **you** have been engaged **so far.**
    a. As **Research team member(s):** Consultants or experts listed as research staff or team members.
    b. Via **Advisory group(s):** Individuals participating in an advisory panel, community advisory board, or other type of discussion group to give input on what to study or how to design the project.
    c. **Via Opinion poll(s) or interview(s):** Individuals complete a set of questions to give input on the research process.
    d. **Other:** Please describe:

    12B. If 12 = Research team member: Were you listed as a co-investigator (e.g., principal investigator) on the grant application that was funded through SPOR? As a co-investigator you likely would have been asked to provide signatures on the paperwork needed to submit the grant.
    a. Yes
    b. No
    c. I don't know

13. People can be involved in many parts of a research project. Please select all of the parts **you** have been involved in **so far**. Please select all that apply.
    a. Helping decide what the project is about (helping choose the research topics or research questions)
    b. Helping develop the grant that supported this project
    c. Helping choose or design the program or treatments being compared
    d. Helping choose the outcomes that matter to patients (what the study will measure) and how to measure them
    e. Helping plan other aspects (parts) of study design – *Please describe*
    f. Helping recruit participants into the study and/or developing ways to ensure participants stay in the study
    g. Helping collect data from study participants
    h. Helping analyze or review results
    i. Helping explain or apply results to real world settings and/or share study findings with those who can use them
    j. Other parts of the project – *Please describe*.

14. How was it decided what parts of the research project you were involved in?

15. Are/were you the only patient partner working with on this research project?
    a. Yes. It was just me → skip to question 17
    b. No. *You and how many other patient partners are/were working together on this research project*
    c. I don't know

16. Are/were all of the patient partners involved in the same parts of the research project?
    a. Yes.
    b. No. *How was it decided what parts different patient partners were involved in?*
    c. I don't know

17. **For each part of the research project selected in question 13, describe what you did and how it influenced (made a difference) to decisions or processes related to this project.** You can fill in as much or as little of this table as you like.

**Project part involved in**	**What did you do?**	**How did this influence (make a difference to) decisions or processes related to this project?**
Example: Choosing the research topics or the medical questions to study	*I explained to others on the research team what research questions matter most to patients*	*The research questions were changed to include the questions important to patients*
Helping decide what the project is about		
Helping develop the grant that supported this project		
Helping choose or design the program or treatments being compared		
Helping choose the outcomes that matter to patients (what the study will measure and how to measure them)		
Helping plan other aspects (parts) of study design (as described in question 13)		
Helping recruit participants into the study and/or developing ways to ensure participants stay in the study		
Helping collect data from study participants		
Helping analyze or review results		
Helping explain or apply results to real world settings and/or share study findings with those who can use them		
Other (as described in question 13)		

18. For each part of the research project selected in question 13, **how much influence** did you have on the decisions that were?

Here are the definitions we use for the types of influences patient partners may have on decision-making:

• **None:** Researcher(s) described decisions to patient partners after decisions were made.
• **Consulted:** Patient partners provide input to researchers that may inform decision-making.

• **Collaborated:** Patient partners work directly with the researcher, in active partnership, to ensure that their perspectives are incorporated in decision-making.
• **Patient/Stakeholder Direction**: Also known as "user control," patient partners have control over the research process and the final decision-making.

**Project part involved in**	**Influences on decision making**				
	**None**	**Consulted**	**Collaborated**	**Patient direction**	**Don't know whether I influenced this part**
Helping decide what the project is about					
Helping develop the grant that supported this project					
Helping choose or design the program or treatments being compared					
Helping choose the outcomes that matter to patients (what the study will measure and how to measure them)					
Helping plan other aspects (parts) of study design (as described in question 13)					
Helping recruit participants into the study and/or developing ways to ensure participants stay in the study					
Helping collect data from study participants					
Helping analyze or review results					
Helping explain or apply results to real world settings and/or share study findings with those who can use them					
Other (as described in question 13)					

19. When thinking of the project overall, how much do you feel…

	None	*A small amount*	*A moderate amount*	*A great deal*	*I don't know*	*Comments (if you have any)*
The patient engagement process integrated a diversity of patient perspectives						
The research that was undertaken reflected the contributions of patient partners						
Patient partners were provided with the support and flexibility they needed to contribute fully to discussions and decisions						
The environment in which the patient engagement activities were set felt safe						
The environment in which the patient engagement activities were set promoted honest interactions						
The environment in which the patient engagement activities were set promoted cultural competence						
Patient partners were provided with						

	None	*A small amount*	*A moderate amount*	*A great deal*	*I don't know*	*Comments (if you have any)*
the training and/or education they needed to contribute fully to discussions and decisions						
Patient partners were provided with adequate financial compensation for their involvement						
Patient partners and researchers acknowledged and valued each others' expertise and experiential knowledge						
Patients, researchers and practitioners work together from the beginning to identify problems and gaps, set priorities for research and work together to produce and implement solutions						
Research is informed and co-directed by patients						
Patient partners and researchers share the goal of timely implementation of quality research						

20. Have the project's results (findings) been disseminated (shared with those who can use them)?
    a. Yes – please describe how
    b. No
    c. Don't know
    d. Not applicable – we don't know our project's findings yet!

21. Do you think that the results (findings) of the project may lead to improved health outcomes?
    a. Yes – *please describe how*
    b. No
    c. Don't know

22. Do you think that the results (findings) of the project may lead to an enhanced healthcare system?
    a. Yes – *please describe how*
    b. No
    c. Don't know

A little about you

23. In what year were you born?

24. What gender do you identify with?
    a. Male
    b. Female
    c. Other (please describe)
    d. Prefer not to answer

25. How do you best describe your ethnic background? If mixed race or other, please specify.
    a. White/Caucasian/European
    b. Black/African-American
    c. East Asian (e.g., Chinese, Korean, Vietnamese)
    d. South Asian (e.g. East Indian, Pakistani, Sri Lankan)
    e. First Nations/Inuit/Metis
    f. Hispanic/Latino/South American
    g. Mixed Race (please specify)
    h. Other (please specify)

26. What is the highest level of education you have completed?
    a. Primary school
    b. Completed secondary school
    c. Completed trade/technical school or college diploma

   d. Completed university degree
   e. Completed graduate degree

27. What province/territory do you live in?
    a. Alberta
    b. British Columbia
    c. Manitoba
    d. New Brunswick
    e. Newfoundland and Labrador
    f. Nova Scotia
    g. Ontario
    h. Prince Edward Island
    i. Quebec
    j. Saskatchewan
    k. Northwest Territories
    l. Nunavut
    m. Yukon

28. What (if any) previous experience do you have with patient engagement in research
    a. No previous experience
    b. Some previous experience (*please describe)*

29. Before partnering on **this** project, had you contributed to a research study as a patient partner?
    a. Yes → How many studies had you been involved with as a patient partner prior to this one?
    b. No

    29B. If 29 = b then:
    Please describe any resources that you relied upon/reached out to for help with being a patient partner for the first time.

30. Have you worked with this study's Primary Applicant(s) ("main researcher(s)"/"leader(s)") on a previous research project?
    a. Yes – as a patient partner
    b. Yes – as a study participant
    c. Yes – in a role other than patient partner or participant (*please describe*)
    d. No

31. What primary community do you represent:
    a. Patient/consumer
    b. Caregiver
    c. Other (*Please describe)*:

Wrap-up

32. Your views are very valuable to us. We welcome any additional thoughts, information, or stories about your experience with this research project.

33. Are you interested in reviewing preliminary findings?
    a. Yes
    b. No

34. Would you like to receive a summary of the study's findings?
    a. Yes
    b. No

35. We would like to conduct follow-up phone interviews with the patient partners that completed this survey. These interviews are being co-led by one of our study's patient partners. Are you interested in learning more about these phone interviews and/or participating in them?
    a. Yes
    b. No

36. Based on the suggestions of our patient partners, our research team is developing an online portal that aims to make it easier for researchers and patient partners with similar research interests to find each other. Would you like to learn more about this online portal, including how you can get involved?
    a. Yes
    b. No

37. As a thank you for your time, would you like to be receive a $5 electronic gift card to Tim Hortons **OR** alternatively have us donate $5 to the Canadian Cancer Society on your behalf?
    a. $5 Tim Hortons gift card
    b. $5 Donation to the Canadian Cancer Society

Please provide your name, email or mailing address below if you answered yes to any of the above questions (32-37). If you wish to receive a gift card, please provide your email address as the gift cards will be distributed electronically to your email address.

This concludes our survey. Thank you for your time!
