# Supplementary material for: Activities and impacts of patient engagement in CIHR SPOR funded research: a cross-sectional survey of academic researcher and patient partner experiences
Source: Res Involv Engagem. 2022 Aug 29;8:44. doi: 10.1186/s40900-022-00376-4 (PMC9423700; doi:10.1186/s40900-022-00376-4)
Supplement: Supplementary file 2 — Additional file 2. Survey of engagement-related activities and impacts: Academic researcher version. [file 40900_2022_376_MOESM2_ESM.pdf]

Supplementary File 2

Please note, as described in our manuscript, this survey contains modified items drawing from PCORI's evaluation of patient engagement in research (i.e., Ways of Engaging-ENgagement ACtivity Inventory Tool (We-ENACT)) and newly created items that measured the elements identified within SPOR Patient Engagement Framework, participants' sociodemographic characteristics and characteristics of their SPOR-funded project.

Survey items were distributed across 8 pages. Technology-assisted completeness checks were not performed prior to survey submission.

Overview

Welcome! The purpose of this online survey is to gather information about the activities and impacts of patient engagement in research projects funded through Strategy for Patient-Oriented Research (SPOR). This online survey should take approximately 15-20 minutes to complete and your participation is entirely voluntary.

Thanks again for your time!

Patient engagement in research: Who, how, and why

You may be involved with more than one project that is funded through a SPOR funding call. However, we sent you sent this link because of your involvement in the project entitled "[project name]". **Please answer the following questions with only this project in mind**.

Note: If you would like to provide answers specific to a different SPOR-funded project that also lists you as a principal investigator on its underlying grant, feel free to do so. But please let us know of the switch by specifying the name of the project that you have chosen to report on below:

2A. Were you listed as a principal investigator on the grant application that resulted in the funding of this project (through a SPOR funding call)? SPOR funding calls took places between 2014 and 2019.

a. Yes → Skip to question 2B
b. No → If you were a **patient partner** (instead of a principal investigator) on a SPOR-funded project, please click on the following link to be taken to the principal investigator version of the survey:

[insert link]

Otherwise, thank you for your interest in our survey but you do not meet our study's eligibility criteria.

Patients can contribute to research projects in many ways. This could include:

- Making sure researchers know what kind of information is important to patients;

- Deciding what the study should be about;
- Deciding who to include in the study;
- Choosing what outcomes the study will measure;
- Tracking study progress;
- Sharing study findings.

We call contributing to research projects in ways like this being a "**patient partner**".

If you were a patient partner in a SPOR funded project, please click on the following link to be taken to the correct version of the survey:

[insert link]

2B. Did this project engage patients as patient partners?

   a. Yes → Skip to question 3.
   b. No → Skip to question 2B1.
   c. I'm uncertain about the extent of patient engagement because I stopped being involved in the project at some point after the grant application was submitted. → Skip to the "A little about you section"

   2B1. Engagement may not occur at all time points or may not occur as planned. Which option best describes why engagement with patient partners has not occurred on the project(s)?

   a. Not part of the research plan
   b. Plans for engagement have been delayed or changed
   c. Other: *(Please describe)*

** those answering question 2B1 will be skipped to the "A little about you section" **

3. What SPOR funding call supported this project?

a. Catalyst Grant: SPOR Innovative Clinical Trials
b. SPOR Networks in Chronic Disease
c. Patient-Oriented Research Collaboration Grants
d. Operating Grant: SPOR Primary and Integrated Health Care Innovations (PIHCI) Network - Programmatic Grants
e. Operating Grant: SPOR PIHCI Network - Quick Strikes
f. Operating Grant: SPOR PIHCI Network - Knowledge Synthesis Grants
g. Operating Grant: SPOR PIHCI Network - Comparative Program and Policy Analysis
h. Operating Grant: SPOR Innovative Clinical Trial (iCT) Multi-Year Grant
i. Operating Grant: SPOR - Guidelines and Systematic Reviews
j. Operating Grant: SPOR & JDRF - iCT Multi-Year Grant
k. Operating Grant: SPOR & JDRF - iCT Multi-Year Grant - Metabolic Control & Artificial Pancreas and Devices
l. Operating Grant: SPOR iCT Rewarding Success - Development Grants
m. Team Grant - SPOR iCT Rewarding Success - Phase 3

n. Other - *Please describe*
o. I don't know

4. Which of the following statement best describes your awareness and involvement in patient engagement activities that occurred in this project?
a. I did not know about and was not involved with the patient engagement activities
b. I somewhat knew of, but was not directly involved with the patient engagement activities
c. I helped plan the patient engagement activities but was not directly involved with them
d. I did not help plan the patient engagement activities, but was directly involved with them
e. I helped plan and was directly involved with the patient engagement activities
f. Other – *Please describe*

5. Other than being a requirement of the funding call, why did this project decide to engage patients as research partners?

6. Please describe how you recruited patient partners to the project.

7. Were patient partners compensated for their involvement in the project? *Select all that apply.*
a. Yes – financially (*please describe)*
b. Yes – other (*please describe*)
c. No
d. I don't know

8. How were patient partners engaged in this project? *Select all that apply.*
a. As **Research team member(s):** Consultants or experts listed as research staff or team members.
b. Via **Advisory group(s):** Individuals participating in an advisory panel, community advisory board, or other type of discussion group to give input on what to study or how to design the project.
c. **Via Opinion poll(s) or interview(s):** Individuals complete a set of questions to give input on the research process.
d. **Other:** Please describe:

8B. If 8 = Research team member: Were any of the patient partners listed as principal investigators on the SPOR-funded grant application?
a. Yes
b. No
c. I don't know

9. Some of the projects funded by SPOR consist of multiple studies. Does the project you are describing in this survey consist of…

a. A single study?

   i. *Please state the study design (e.g., scoping review, cross-sectional study, randomized controlled trial)*

   ii. What research phase is this project in?

      a. Planning
      b. Recruitment
      c. Data collection
      d. Data analysis/reviewing results
      e. Writing-up/disseminating results
      f. The project is done
      g. Other – *Please describe*
      h. I don't know

b. Multiple studies? *Please state how many studies in total comprised the project. How many of the studies engaged patient as partners? Please name each of the project's studies that engaged patients are partners by their study design (one per box).*

9B. If 9 = b (multiple studies): Did the ways in which patient partners were engaged vary between the project's studies?

   a. Yes
   b. No

If the answer to Question 9B = a (yes), then the following text will pop up prior to question 10:

Questions 10-15 should be answered in relation to the study that you labelled as [insert label]

10. What research phase is this study in?

   i. Planning
   j. Recruitment
   k. Data collection
   l. Data analysis/reviewing results
   m. Writing-up/disseminating results
   n. The project is done
   o. Other – *Please describe*
   p. I don't know

11. **In what stage(s)** of this study were patient partners engaged? *Select all that apply.*

a. Deciding what the project is about (helping direct the research topics or the research questions)
b. Involvement in grant development
c. Choosing or designing interventions or comparators
d. Choosing outcomes and deciding how to measure them
e. Other aspects of study design – *Please describe*
f. Recruiting or retaining study participants

g. Data collection
h. Analyzing or reviewing results
i. Dissemination
j. Other part of the project - *Please describe:*

12. How was it decided what stage(s) of the project patient partners were engaged in?

13. For each stage of the project (or specific study) that patients were engaged (as selected in question 11), which choice best reflects the ways in which patient partners were engaged?

Definitions:
• **Information:** Researcher(s) describe decisions to patient partners after decisions are made.
• **Consultation:** Patient partners provide input to researchers that may inform decision-making.
• **Collaboration:** Patient partners work directly with the researcher, in active partnership, to ensure that their perspectives are incorporated in decision-making.
• **Patient/Stakeholder Direction**: Also known as "user control," patient partners have control over the research process and the final decision-making.

STAGE	*Information*	*Consultation*	*Collaboration*	*Patient/Stakeholder*	*Don't know*
a. Deciding what the project is about					
b. Involvement in grant development					
c. Choosing or designing interventions or comparators					
d. Choosing outcomes and deciding how to measure them					
e. Other aspects of study design (as described in question 11)					
f. Recruiting or retaining study participants					
g. Data collection					
h. Analyzing or reviewing results					
i. Dissemination					

STAGE	*Information*	*Consultation*	*Collaboration*	*Patient/Stakeholder*	*Don't know*
j. Other parts of the project (as described in question 11)					

14. For each stage of the project (or specific study) that patients were engaged (as selected in question 11), describe **what patient partners actually did** and **any impact** this had on the project/research process.

	What patient partners did	*The impact of what patient partners did on the project/research process*
Example: Deciding what the project is about	*Explained to the research team the questions that matter most to patients*	*The research questions were changed to include the questions important to patients*
a. Deciding what the project is about		
b. Involvement in grant development		
c. Choosing or designing interventions or comparators		
d. Choosing outcomes and deciding how to measure them		
e. Other aspects of study design (as described in question 8)		
f. Recruiting or retaining study participants		
g. Data collection		
h. Analyzing or reviewing results		
i. Dissemination		
j. Other parts of the project (as described in question 8)		

If the answer to Question 9B = a (yes), then the following text and question (14B) will pop up prior to question 15:

14B. Did any of the other studies engage patients as partners?

a. Yes → Repeat questions 10-14 with the following text: please think of <u>the second/third/fourth study to engage patients as partners</u> within the project <u>only</u> when answering questions 10-14. We will continue to prompt you to re-answer these questions (11-14) for the other studies that engaged patients as partners separately.

b. No → Go to question 15.

15. When thinking of the project overall, how much do you feel…

	None	*A small amount*	*A moderate amount*	*A great deal*	*I don't know*	*Comments (if you have any)*
The patient engagement						

	None	*A small amount*	*A moderate amount*	*A great deal*	*I don't know*	*Comments (if you have any)*
process integrated a diversity of patient perspectives						
The research that was undertaken reflected the contributions of patient partners						
Patient partners were provided with the support and flexibility they needed to contribute fully to discussions and decisions						
The environment in which the patient engagement activities were set felt safe						
The environment in which the patient engagement activities were set promoted honest interactions						
The environment in which the patient engagement activities were set promoted cultural competence						
Patient partners were provided with the training and/or education they needed to						

	None	*A small amount*	*A moderate amount*	*A great deal*	*I don't know*	*Comments (if you have any)*
contribute fully to discussions and decisions						
Patient partners were provided with adequate financial compensation for their involvement						
Patient partners and researchers acknowledged and valued each others' expertise and experiential knowledge						
Patients, researchers and practitioners work together from the beginning to identify problems and gaps, set priorities for research and work together to produce and implement solutions						
Research is informed and co-directed by patients						
Patient partners and researchers share the goal of timely implementation of quality						

16. Have the results of your research project been disseminated?

a. Yes – *please describe how*
b. No
c. Don't know

d. Not applicable – we don’t know our project’s findings yet!

17. Do you think that the results (findings) of the project may lead to improved health outcomes?
a. Yes – *please describe how*
b. No
c. Don’t know

18. Do you think that the results (findings) of the project may lead to an enhanced healthcare system?
a. Yes – *please describe how*
b. No
c. Don’t know

A little about you

19. In what year were you born?

20. What gender do you identify with?
    a. Male
    b. Female
    c. Other (please describe)
    d. Prefer not to answer

21. How do you best describe your ethnic background? If mixed race or other, please specify.
a. White/Caucasian/European
b. Black/African-American
c. East Asian (e.g., Chinese, Korean, Vietnamese)
d. South Asian (e.g. East Indian, Pakistani, Sri Lankan)
e. First Nations/Inuit/Metis
f. Hispanic/Latino/South American
g. Mixed Race (please specify)
h. Other (please specify)

22. What province/territory do you live in?
a. Alberta
b. British Columbia
c. Manitoba
d. New Brunswick
e. Newfoundland and Labrador
f. Nova Scotia
g. Ontario
h. Prince Edward Island
i. Quebec
j. Saskatchewan

k. Northwest Territories
l. Nunavut
m. Yukon

23. What department/discipline is your primary academic appointment situated in?

24. How many years have you worked as a Primary Investigator?

25. How many successful SPOR funding calls (in total) have you been a part of?

26. Have you worked with your project's patient partners on a previous research project? *Select all that apply*

a. Yes – they were also patient partners
b. Yes – they were study participants
c. Yes – they were in a role other than patient partner or participant (*please describe*)
d. No

27. What (if any) previous experience do you have with patient engagement in research

a. No previous experience
b. Some previous experience (*please describe)*

27B. If 27 = a then:
Please describe any resources that you relied upon/reached out to for help with entering into this research domain.

28. What is your self-reported "comfort" with patient engagement in research?
    a. None
    b. A small amount
    c. A Moderate amount
    d. A great deal

29. Thank you for your responses to the above survey items. Please feel free to share any other information about engagement in your project – challenges, strategies you've identified to facilitate work with research partners, positive and negative impacts on your work

30. We are also interested in the perspectives of the patient partners involved with your project. There are several ways you can support us in this. Please indicate your preference below.

a. Yes – <u>I can provide you with </u>patient partners' contact information now (sent to a different IP location): Please provide patient partners' names and contact information (e.g., email or mailing address).
b. Yes – <u>reach out to me directly</u> and I will get patient partners' contact information to you (sent to a different IP location): Please provide name, email or phone number.

c. Yes – I will pass on your study information to the patient partners involved with this project (e.g., forward the recruitment email you sent me, send them a survey link).
d. No – I cannot connect you with the patient partners involved with this project.

31. Are you interested in reviewing preliminary findings?
a. Yes
b. No

32. Would you like to receive a summary of the study's findings?
a. Yes
b. No

33. Based on the suggestions of our patient partners, our research team is developing an online portal that aims to make it easier for researchers and patient partners with similar research interests to find each other. Would you like to learn more about this online portal, including how you can get involved?
a. Yes
b. No

34. As a thank you for your time, would you like to be receive a $5 electronic gift card to Tim Hortons **OR** alternatively have us donate $5 to the Canadian Cancer Society on your behalf? If so please indicate your preference below:
a. $5 Tim Hortons gift card
b. $5 Donation to the Canadian Cancer Society

Please provide your name, email or mailing address below if you answered yes to any of the above questions (31-34). If you wish to receive a gift card, please provide your email address as the gift cards will be distributed electronically to your email address.

This concludes our survey. Thank you for your time!
