# Supplementary figures and images for: Activities and impacts of patient engagement in CIHR SPOR funded research: a cross-sectional survey of academic researcher and patient partner experiences
Source: Res Involv Engagem. 2022 Aug 29;8:44. doi: 10.1186/s40900-022-00376-4 (PMC9423700; doi:10.1186/s40900-022-00376-4)

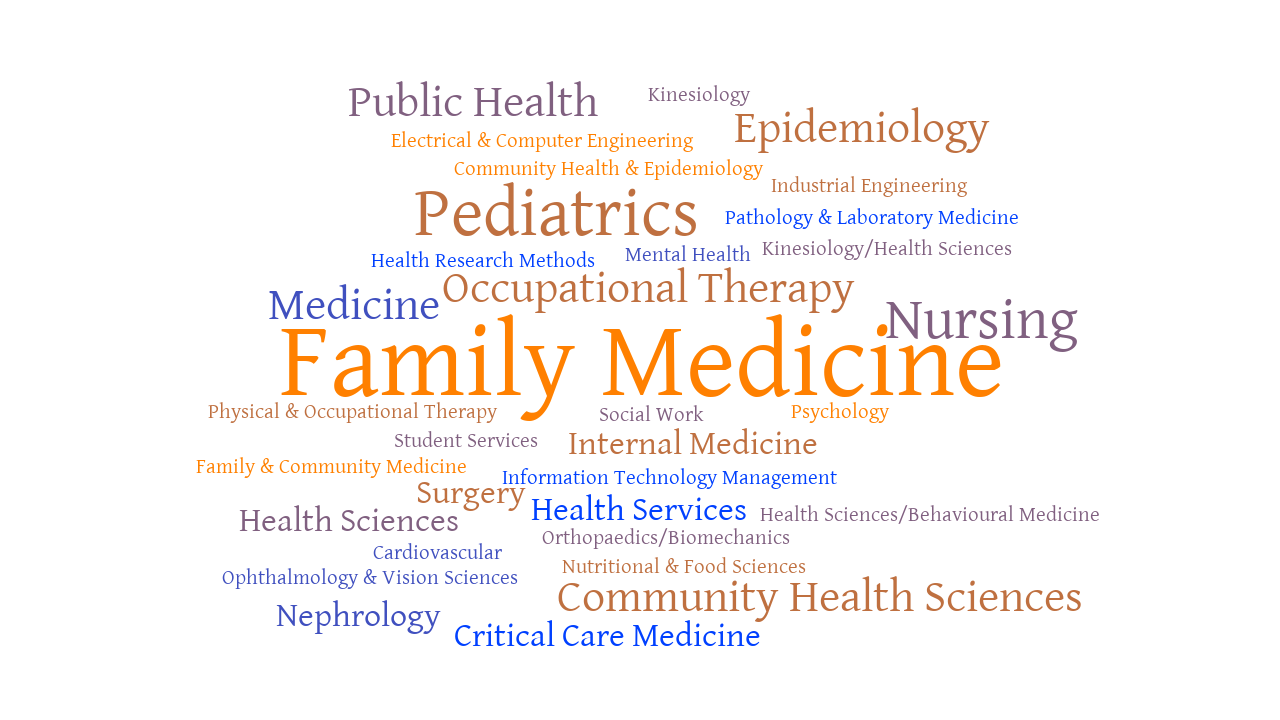

Supplement: Supplementary file 3 — Additional file 3. Wordcloud of academic researcher respondents’ departments/disciplines. [file 40900_2022_376_MOESM3_ESM.png]
