# Supplementary material for: Activities and impacts of patient engagement in CIHR SPOR funded research: a cross-sectional survey of academic researcher and patient partner experiences
Source: Res Involv Engagem. 2022 Aug 29;8:44. doi: 10.1186/s40900-022-00376-4 (PMC9423700; doi:10.1186/s40900-022-00376-4)
Supplement: Supplementary file 4 — Additional file 4. Terms encompassed by each reported impact type. [file 40900_2022_376_MOESM4_ESM.pdf]

Supplementary File 4

1. Created

- Developed
- Initiated
- Designed
- Defined
- Concept emerged
- Enthusiasm led to grant being written

2. Moulded (added/subtracted/changed/shaped/improved)

- Enhanced
- Improved
- Strengthened
- Refined
- Shaped
- Revised
- Suggested
- Added
- Identified themes
- Identified others interested in the topic
- Identified what needed revision
- Changed/changes were made
- Modified
- Broadened
- Informed
- Contributed ideas
- Caught issues

3. Confirmed

- Confirmed
- Supported ideas being proposed

4. Chosen/prioritized

- Selected
- Chose
- Helped achieve consensus
- Helped decide/determine
- Prioritized
- Identified top interventions to be studied

5. Successful

- Contributed to the acceptance of the grant
- Helped secure the grant
- Integral to success

6. Done/carried out

- Participated in
- Prepared data
- Helped analyze data
- Answered participant questions
- Collected data
- Determined if items were gathering intended data
- Validated
- Established feasibility
- Piloted
- Enthusiasm led to project being conducted

7. Limited/no/negative impact

***statements provided in manuscript tables 2-6
